# Transmission potential of *Streptococcus pyogenes* during a controlled human infection trial of pharyngitis

**DOI:** 10.1128/msphere.00513-24

**Published:** 2024-09-10

**Authors:** Stephanie L. Enkel, Bernadette Wong, Thel K. Hla, Janessa Pickering, Timothy C. Barnett, Hannah M. M. Thomas, Nina Lansbury, Jonathan R. Carapetis, Joshua Osowicki, Andrew Steer, Laurens Manning, Asha C. Bowen

**Affiliations:** 1 Wesfarmers Centre of Vaccines and Infectious Diseases, Telethon Kids Institute, University of Western Australia, Nedlands, Western Australia, Australia; 2 Medical School, University of Western Australia, Crawley, Western Australia, Australia; 3 Department of Infectious Diseases, Fiona Stanley Hospital, Murdoch, Western Australia, Australia; 4 Marshall Centre for Infectious Diseases Research and Training, School of Biomedical Sciences, University of Western Australia, Nedlands, Western Australia, Australia; 5 School of Public Health, University of Queensland, Brisbane, Queensland, Australia; 6 Department of Infectious Diseases, Perth Children’s Hospital, Nedlands, Western Australia, Australia; 7 Tropical Diseases Research Group, Murdoch Children’s Research Institute, Melbourne, Victoria, Australia; 8 Department of Paediatrics, The University of Melbourne, Parkville, Victoria, Australia; 9 Infectious Diseases Unit, Department of General Medicine, Royal Children’s Hospital Melbourne, Parkville, Victoria, Australia; 10 Department of General Medicine, Royal Children’s Hospital, Melbourne, Victoria, Australia; The University of Texas Medical Branch at Galveston, Galveston, Texas, USA

**Keywords:** *Streptococcus pyogenes*, environmental transmission of microbes, acute rheumatic fever, rheumatic heart disease, human infection study, infection prevention, infection control, infection transmission

## Abstract

Controlled human infection (CHI) models can provide insights into transmission of pathogens such as *Streptococcus pyogenes* (Strep A). As part of the Controlled Human Infection with Penicillin for *Streptococcus pyogenes* (CHIPS) trial, we explored the potential for transmission among participants deliberately infected with the Strep A emm75 strain. Three approaches to understanding transmission were employed: the use of agar settle plates to capture possible droplet or airborne spread of Strep A; measurement of distance droplets could spread during conversation; and environmental swabbing of high-touch items to detect Strep A on surfaces. Of the 60 (27%) CHIPS trial participants across five cohorts, 16 were enrolled in this sub-study; availability of study staff was the primary reason for selection. In total, 189 plates and 260 swabs were collected. Strep A was grown on one settle plate from a participant on the second day, using plates placed 30 cm away. This participant received the placebo dose of penicillin and had met the primary endpoint of pharyngitis. Whole-genome sequencing identified this to be the challenge strain. Strep A was not detected on any swabs. In this small sample of CHI participants, we did not find evidence of Strep A transmission by the airborne route or fomites, and just one instance of droplet spread while acutely symptomatic with streptococcal pharyngitis. Although these experiments provide evidence of minimal transmission within controlled clinical settings, greater efforts are required to explore Strep A transmission in naturalistic settings.

**IMPORTANCE:**

*Streptococcus pyogenes* remains a significant driver of morbidity and mortality, particularly in under-resourced settings. Understanding the transmission modalities of this pathogen is essential to ensuring the success of prevention methods. This proposed paper presents a nascent attempt to determine the transmission potential of *Streptococcus pyogenes* nested within a larger controlled human infection model.

## INTRODUCTION

*Streptococcus pyogenes* (Strep A) is a human-only bacterial pathogen causing a wide spectrum of disease including superficial (skin and throat infections), invasive (sepsis and skeletal infections), and immune-mediated conditions such as acute rheumatic fever (ARF) and rheumatic heart disease (RHD) (1). Penicillin remains the mainstay of Strep A therapy (2). Globally, there are over 40.5 million cases of RHD causing more than 340,000 annual deaths (3) and 177,000 deaths due to invasive disease (4). Sore throats—Strep A bacterial pharyngitis included—are one of the most common reasons for attending primary health-care services, contributing to significant societal costs including missed school/work days, antibiotic use, and if not treated early, severe downstream consequences (5).

Numerous strands of epidemiological evidence confirm that human-to-human transmission of Strep A occurs between and within families, close contacts, households, and community settings. However, understanding the transmission dynamics during episodes of sore throat is important for clinicians, epidemiologists, public health practitioners, and vaccine developers, particularly when designing and evaluating the impact of different interventions. Experiments performed in the 1940s, where patients with proven Strep A pharyngeal colonization were asked to talk, cough, or sneeze, demonstrated infrequent positive cultures of the pathogen from “settle plates” placed close to the patient (6). These results suggested that spread of Strep A was possible via large respiratory droplets when coughing and sneezing, but not talking (6–10). Identification of Strep A from elevated settle plates collected from London schools during a recent scarlet fever outbreak raises the possibility of airborne spread, via particles smaller than respiratory droplets (11). The survival of Strep A in laboratory-derived aerosols also supports observational data but suggests that high humidity may be an additional critical factor to facilitate airborne transmission (12), while research has implicated surfaces as having a role in transmission events via fomites (13, 14).

Controlled human infection (CHI) models can provide insights into transmission of pathogens such as Strep A. The intentional exposure of healthy volunteers to pathogens in a regulated environment has been increasingly applied in medical and health research in the past few decades as an important avenue toward accelerating the development of vaccines and other therapeutic options. All recent, validated CHI models are guided by strict ethical frameworks and independent review to assess benefits and risks, with participant safety upheld as a priority (15).

A validated Strep A CHI model was recently established using an extensively characterized strain of *emm*75 Strep A (M75), reliably inducing acute streptococcal pharyngitis in more than 60% of healthy adult volunteers (16). In the Controlled Human Infection with penicillin for Strep A [the Controlled Human Infection with Penicillin for *Streptococcus pyogenes* (CHIPS) trial (ACTRN12621000751875)], we adapted this model to investigate the lowest concentration of penicillin required to prevent pharyngitis, with the ultimate aim of informing new and improved formulations of long-acting penicillins for the secondary prophylaxis of ARF/RHD (17). The controlled settings of the study provided an opportunity to explore the potential for transmission among participants deliberately infected with Strep A M75 strain and to observe the potential for between-participant transmission during future Strep A CHI experiments.

## MATERIALS AND METHODS

The CHIPS trial (17) was designed to determine the lowest plasma concentration of penicillin needed to prevent experimental human streptococcal pharyngitis, with 60 participants randomized to receive intravenous infusions of penicillin at steady-state concentrations ranging from 0 (placebo) to 20 ng/mL. After at least 12 hours into the infusion, a throat swab with an inoculum of *emm75* Strep A was directly applied to the pharynx (challenge procedure) (16, 18). Participants were then admitted and monitored in an inpatient facility for up to 6 days for the development of acute symptomatic pharyngitis. Throat swabs were taken every 12 hours from all participants regardless of symptoms to identify pharyngeal colonization.

### Study procedures

**Fig 1:**
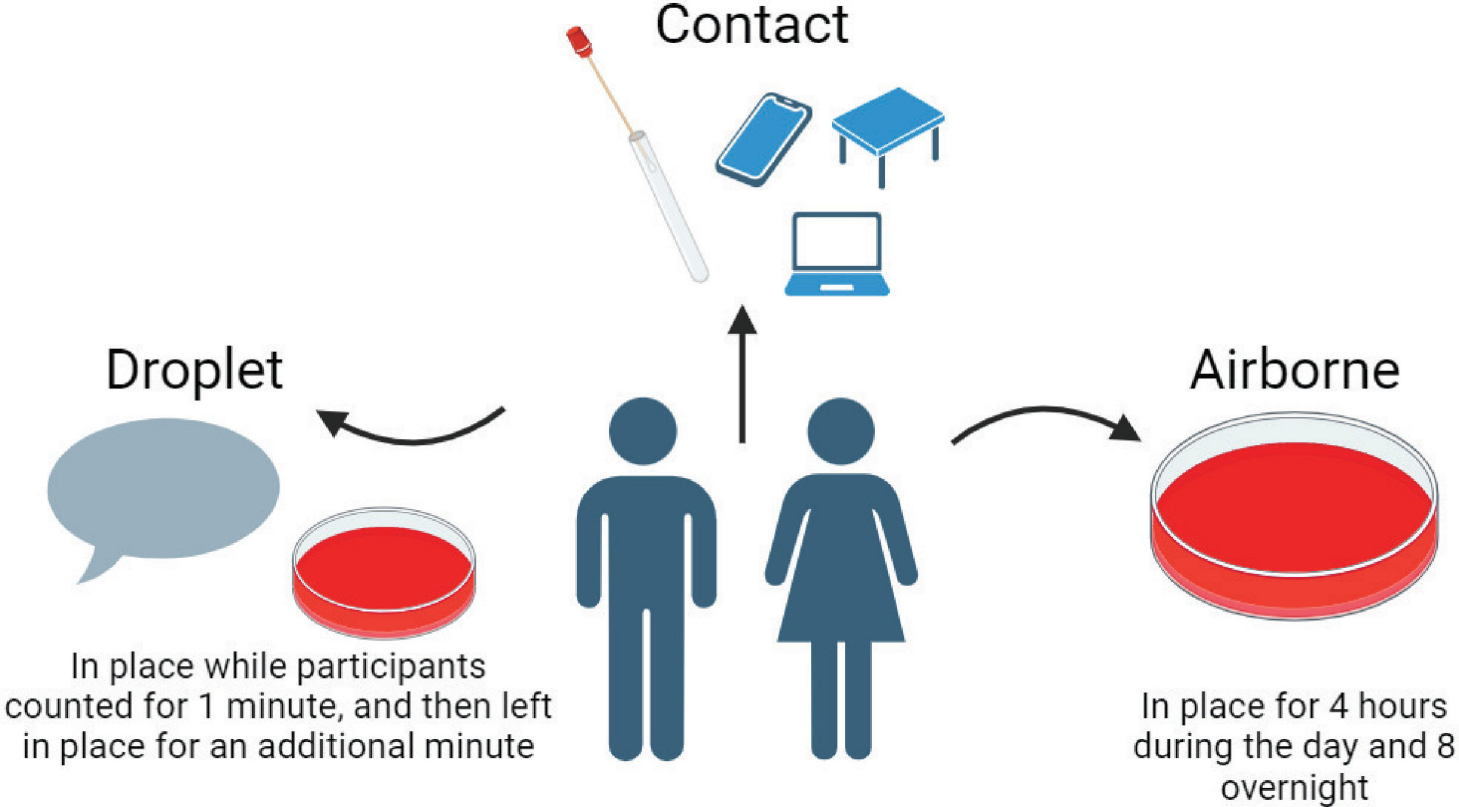
Three unique experiments were conducted during the study: (i) droplet, (ii) contact, and (iii) airborne.

The approach to assessing airborne, fomite, and droplet transmission potential of Strep A in three experiments has previously been described in detail (Fig. 1) (19).

The first CHIPS trial participants were challenged in September 2022. The selection of cohorts involved in this sub-study was primarily informed by availability of study staff to complete the nested experimentation, which were completed between October 2022 and April 2023. In brief, airborne and droplet transmission were examined through the placement of selective settle plates (horse blood agar containing colistin and nalidixic acid HBA-CNA; PathWest Laboratories, Western Australia) 2 m above the floor in each participant inpatient cubicle and another on their overbed table at a height of approximately 90 cm. These remained in place between the hours of 09:00 and 13:00 on Day 2 (from 24 hours post-challenge) and Day 3 (from 48 hours post-challenge). HBA-CNA plates were also placed in these positions overnight between 22:00 on Day 2 and 06:00 on Day 3 while participants slept.

Secondly, the distance that Strep A traveled from a participant with suspected infection was studied at two timepoints: 24 hours post-challenge and 48 hours post-challenge, commencing at 09:00 on Days 2 and 3, respectively. Participants were seated on their beds in front of a table adjusted to chest height, with a settle plate placed 30 cm from their chest. They were then asked to count upward from “1” for 1 minute using a conversational tone, after which the plate was left in place for another minute. This process was repeated with new plates placed 90 and 180 cm from each participant.

Lastly, surfaces through the participant’s room were swabbed as well as five frequently touched personal items. Swabbing of common-area objects was determined by observation of high-touch items during the morning participant checks by clinical staff and included medical equipment, face shields, shared tables, light switches, trolleys, communal water jugs and curtains. For each participant, the following were swabbed: bed remote control, bedside table, water cup, intravenous stand, and a personal item (Table 1). This was done twice, immediately after completion of the droplet transmission experiments. A surface area of approximately 25 cm^2^ for each item was swabbed in three directions (Copan regular flocked swab; Copan, Italy) and placed immediately in a cryovial containing 0.5 mL of skim milk, glucose, and glycerol broth solution (PathWest Laboratories, Western Australia).

**TABLE 1:** Participants and sample collection

	Cohort 1		Cohort 2			Cohort 3			Cohort 4			Cohort 5				
Dates of nested experimentation (day/month/year)	12–13/10/2022		26–27/10/2022			7-8/12/2022			15–16/03/2023			19–20/04/2023				
Participant number	1	2	3	4	5	6	7	8	9	10	11	12	13	14	15	16^*a*^
Met CHIPS endpoint of pharyngitis (Y/N)^*b*^	N	N	N	N	Y	N	N	Y	N	N	Y	N	N	N	N	Y
Met colonization endpoint by D5 (qPCR^*b*^ Ct <30, Y/N)	N	Y	N	Y	Y	N	N	Y	Y	Y	Y	N	N	N	N	Y
+ve throat swab (Y/N)	N	Y—D5	N	Y—D5	Y—D4	N	N	Y—D4	Y—D5	Y—D5	Y—D3	N	N	N	N	Y—D3
Assigned penicillin concentration (ng/mL)	20	3	12	12	0	20	6	3	3	6	0	12	3	6	20	0
Actual penicillin concentration (mean, ng/mL)	20.4	1.6	11.9	13.5	0.0	20.1	10.5	2.4	1.5	6.4	0.0	14.1	2.8	7.2	24.3	0.0
+ve environmental samples (Y/N)	N	N	N	N	N	N	N	N	N	N	**Y—30** cm^*c*^	N	N	N	N	N
Number of plates	24 (12 droplet, 12 airborne)		36 (18 droplet, 18 airborne)			36 (18 droplet, 18 airborne)			36 (18 droplet, 18 airborne)			57 (27 droplet, 30 airborne)				
Number of swabs (confinement room)	20		20			20			20			20				
Number of swabs (personal)	20		30			30			30			50				
Personal item swabbed	Phone	Game device	PC mouse	Laptop	Laptop	Head phones	Game device	Laptop	Head phones	Book	Laptop	Head phones	Laptop	Phone	Painting cup	Notebook

^*a*^ Did not consent to transmission experiments on Day 3 due to pharyngeal pain.

^*b*^ Quantitative polymerase chain reaction.

^*c*^ Had lost voice by Day 3 so coughed during transmission experiments instead of counting to 100.

Discussions during the design phase of this sub-study with the contract research organization (CRO) eliminated the opportunity to ask all participants to cough or induce sneezing due to infection control concerns amid the coronavirus disease 2019 (COVID-19) epidemic. In Western Australia where this research was conducted, border closures until March 2022 kept COVID-19 rates extremely low; however, disease incidence was still high in September 2022 when the first participant was challenged. For these reasons, the CRO was highly cautious to possible unintended transmission of COVID-19, and the research team was responsive to their policies.

Standard infection control measures were in place for prevention of COVID-19 at the facility, including staff and participants wearing masks. All participants and staff were required to wear surgical masks or particulate filter respirators (except during experiments, sample collection, and sleep). Additionally, a high-efficiency particulate air (HEPA) filter (Samsung model AX90T7080WD) remained in use in the participants’ environment throughout the trial.

### Microbiological and statistical analysis

Environmental swabs and HBA-CNA plates were refrigerated (4°C–8°C) prior to transport to the laboratory at Telethon Kids Institute (Perth, Western Australia). Swabs were then cultured for beta-hemolytic streptococci according to Clinical and Laboratory Standards Institute methods (20). The presence of *S. pyogenes* as indicated by β-hemolytic morphology was confirmed with sub-culture, bacitracin sensitivity testing, and positive group A latex agglutination reaction (Streptex, Thermo Scientific). The binary outcome for this nested sub-study was defined as presence or absence of the Strep A challenge strain in cultures of collected samples.

Investigators were blinded to the penicillin dose level each participant received until all data were collected. Quantitative polymerase chain reaction (qPCR) methods were completed on daily throat swabs taken from participants with Ct of <30 as the chosen threshold to indicate presence of Strep A. Whole-genome sequencing (WGS) was completed for any identified Strep A colonies. Briefly, isolates were sequenced on an Illumina NovaSeq platform, and sequences were matched to reference strains. The definitions of confirmed pharyngitis and colonization for participants are provided elsewhere (21).

## RESULTS

Sixteen CHIPS trial participants were involved in this sub-study across five occasions, during which ambient temperature was kept between 22°C and 24°C. Humidity was not recorded. In total, 189 plates and 260 swabs were collected. A light growth (one to two colonies) of Strep A was detected on only one HBA-CNA plate placed at 30 cm from participant 11, who was symptomatic with pharyngitis (hoarse voice on Day 2 and cultured Strep A from pharyngeal swabs on Day 3).With permission of the CRO, this participant elected to cough instead of talk during the second day of transmission experimentation. Participant 11 was assigned to a placebo dose of penicillin. WGS identified this to be the challenge strain (*emm*75). Strep A was not detected on any environmental swabs (Table 1).

Four of the 16 participants met the primary endpoint of acute pharyngitis with two of these on Day 3 while experiments were being performed. Participant 16 (cohort 5) chose not to participate in the second day of experiments due to discomfort related to pharyngitis.

## DISCUSSION

In this controlled human infection challenge sub-study, we found little evidence of Strep A in the environment and risk of transmission from droplet, airborne, or surfaces among participants with Strep A pharyngitis or pharyngeal colonization. We detected Strep A in a close-range experiment from a single patient, suggesting droplet spread in this instance. Strep A was not detected in experiments designed to explore transmission by airborne and fomite routes. However, it is important to note that despite limited evidence of transmission, these experiments occurred in a tightly controlled, air-filtered environment where most participants were asymptomaticand .

There has been little advancement in understanding of Strep A transmission since experiments conducted in the 1940s by Hamburger and Robertson (6), which informed our experimental design in this study. They invited participants with proven *S. pyogenes* pharyngeal colonization to talk and induced coughing and sneezing while settle plates at different distances captured possible droplet spread. It is unlikely that infection control practices including surface cleaning, HEPA filtration, and mask use were part of the experimental design. “Broth air samplers” (6) were also used but did not identify Strep A. The authors concluded that coughing and sneezing could discharge large quantities of Strep A, but this was an infrequent finding among the Strep A colonized participants. Talking was noted as expelling negligible Strep A particles; participants also counted for a total of 5 minutes with plates at different distances in place throughout this period. We did not elect to use contemporary air samplers due to the better results from settle plates in earlier studies but would include these in future studies. Further and as explained in Materials and Methods, we were unable to widely explore transmissibility of Strep A following coughing and sneezing due to strict infection control policies in place by the CRO.

Oswin et al. have recently measured the airborne stability of Strep A using the Controlled Electrodynamic Levitation and Extraction of Bioaerosols onto a Substrate instrument (12). Their results highlighted the importance of humidity, with 50%–80% of exhaled Strep A particles remaining viable for at least 5 minutes when humidity exceeded 70% (12). It is conceivable that infection control measures during the CHIPS trial, including HEPA filtration and consistent airconditioned indoor temperature and reduced humidity, could have limited the duration of Strep A airborne stability. In more domiciliary settings, airborne transmission may be more likely. As Oswin et al. (12) and Cordery et al. (11) recommend, further experimentation in different environmental conditions is needed.

The stringent safety requirements of human challenge studies also extend to the protection of research staff from the infectious agent being studied. In this study, droplet transmission was confirmed in only 1 of 93 (1.2%) experiments. The limited transmission is a reassuring safety signal for staff undertaking future Strep A pharyngitis human challenge research.

Several aspects of this nested study limit the application of our findings. Infection prevention and control strategies, bolstered in the context of the COVID-19 pandemic, and the climate-controlled inpatient clinical setting—inclusive of HEPA filtration—may have prevented airborne or droplet transmission. However, settle plates exposed for a prolonged period during mask-free sleep time also failed to detect Strep A. The sample size was also relatively small and included some participants who did not have pharyngitis and others who had neither established infection nor colonization. It is likely that the high proportion of participants receiving penicillin at any dose resulted in suppression of pharyngeal Strep A. Of the 16 participants in the sub-study, four developed the primary endpoint, with just one producing a positive environmental sample, while coughing rather than talking as planned. Transmission of Strep A via droplets (and coughing) is already well documented (6–8). Based on the CHIVAS-M75 trial, where all participants were symptomatic by Day 2 of experimentation, the penicillin challenge may also have delayed symptoms and culture positivity with sampling occurring to match the likely peak of Strep A infection. However, these noted limitations did not influence the ability to detect Strep A from any of the transmission routes under investigation.

It is also conceivable that more sensitive molecular methods may have detected Strep A where culture did not; however, it is unclear whether molecular detection can result in transmission. While culture remains the “gold standard” for clinical detection of Strep A from throat swabs, highly sensitive molecular methods have been developed for clinical and research use to detect and quantify Strep A, with a lower limit of detection than culture (22–24). The use of settle plates is still conventionally accepted as an effective method to detect Strep A in the environment—specifically for airborne and droplet transmission—and is used in several foundational studies that informed this research (11, 25–27). Future studies could enhance environmental detection using settle plates by using enrichment media (e.g., Todd-Hewitt broth with 1% yeast extract) (28) or molecular methods, such as qPCR (29) or qualitative (present/absent) molecular point-of-care-test platforms (30), although the transmission potential of Strep A below the limit of detection by culture is uncertain. Overly sensitive qPCR methods may also detect non-viable Strep A that cannot be transmitted; hence, culture remains the preferred modality.

### Conclusion

In this small sample of human challenge trial participants, we did not find evidence of Strep A transmission potential by the airborne route or fomites and just one instance of potential for droplet spread when a participant was asked to cough. This result indicates a need for future study protocols to include coughing or sneezing experimentation. Although these experiments are reassuring for efficacy of infection control measures, greater efforts are required to explore Strep A transmission in households and classrooms. While a human challenge model could conceivably be modified for such a purpose, focusing on participants with acute symptomatic pharyngitis, no exposure to treatment and modifying environmental conditions such as humidity, a simpler pathway may be to repeat these experiments using more sensitive, high-throughput (e.g., qPCR) or molecular point-of-care tests in children and adults with sore throat in the community.
